# Increased citrullination and expression of peptidylarginine deiminases independently of *P. gingivalis* and *A. actinomycetemcomitans* in gingival tissue of patients with periodontitis

**DOI:** 10.1186/s12967-018-1588-2

**Published:** 2018-07-31

**Authors:** Marianne Engström, Kaja Eriksson, Linkiat Lee, Monika Hermansson, Anders Johansson, Anthony P. Nicholas, Natalija Gerasimcik, Karin Lundberg, Lars Klareskog, Anca Irinel Catrina, Tülay Yucel-Lindberg

**Affiliations:** 1 Rheumatology Unit, Department of Medicine, Karolinska Institutet, Stockholm, Sweden; 2 Department of Dental Medicine, Karolinska Institutet, Huddinge, Sweden; 3 Division of Molecular Periodontology, Department of Odontology, Umeå University, Umeå, Sweden; 4 Department of Neurology, University of Alabama at Birmingham and Birmingham VA Medical Center, Birmingham, AL USA

**Keywords:** *Aggregatibacter actinomycetemcomitans*, Citrullinated proteins, Gingival tissue, Inflammation, Leukotoxin, Peptidylarginine deiminases, Periodontitis, *Porphyromonas gingivalis*

## Abstract

**Background:**

A relationship between rheumatoid arthritis (RA) and periodontitis has been suggested from findings that individuals with RA are prone to have advanced periodontitis and vice versa. In search of possible common pathogenetic features of these two diseases, we investigated the presence of citrullinated proteins and expression of endogenous peptidylarginine deiminases **(**PAD2 and PAD4), in periodontal tissue of individuals with periodontitis and healthy controls, in relation to the periodontal pathogens *Porphyromonas gingivalis* (*P. gingivalis*) and *Aggregatibacter actinomycetemcomitans* (*A. actinomycetemcomitans*), producing leukotoxin as virulence factor. These two oral bacteria have been suggested to be linked to anti-citrullinated protein antibodies in patients with RA.

**Methods:**

Gingival tissue biopsies were obtained from 15 patients with periodontitis and 15 individuals without periodontal disease. Presence of CD3-positive lymphocytes, citrullinated proteins, PAD2, PAD4, *P. gingivalis* as well as *A. actinomycetemcomitans* and *Mannheimia haemolytica* produced leukotoxins were analysed by immunohistochemistry, followed by triple-blind semi-quantitative analysis. Mann–Whitney and Fisher’s exact tests were used to analyse differences between groups. *PADI2* and *PADI4* mRNA levels were assessed by RT-qPCR and analysed using Wilcoxon signed rank test.

**Results:**

Increased staining of citrullinated proteins was observed in gingival connective tissue from subjects with periodontitis (80%, 12/15) compared to healthy gingival tissue (27%, 4/15), whereas no differences were observed in gingival epithelium. There was also an increased staining of the citrullinating enzymes PAD2 and PAD4 in gingival connective tissue of patients with periodontitis whereas similar levels of PAD2 and PAD4 were observed in the gingival epithelium of the two groups. Similarly, the mRNA levels of *PADI2* and *PADI4* were also increased in the gingival tissue of patients with periodontitis compared to healthy controls. Furthermore, presence of *P. gingivalis* and leukotoxins was comparable in both epithelium and connective tissue, from the different investigated individuals with and without periodontitis, and there were no correlations between the presence of periodontal pathogens and the expression of citrullinated proteins or PAD enzymes.

**Conclusion:**

Chronic gingival inflammation is associated with increased local citrullination and PAD2 and PAD4 expression in periodontitis. The increased citrullination and PAD2 and PAD4 expression in periodontitis were, however, independent of the presence of periodontal pathogen *P. gingivalis* and *A. actinomycetemcomitans* leukotoxin.

**Electronic supplementary material:**

The online version of this article (10.1186/s12967-018-1588-2) contains supplementary material, which is available to authorized users.

## Background

Periodontitis is a major cause of tooth loss and one of the most prevalent chronic infectious inflammatory diseases, affecting up to 46% of the adult population [1, 2]. The disease share several common features, including genetic association to human leukocyte antigen (HLA) DR4 alleles and smoking as environmental risk factors with rheumatoid arthritis (RA), another chronic inflammatory disease [3, 4]. One of the potential links between these two diseases is the oral pathogen *Porphyromonas gingivalis* (*P. gingivalis*), Gram-negative anaerobic bacteria associated with periodontitis. This pathogen was proposed to possibly contribute to the generation of citrullinated antigens and the production of citrullinated protein antibodies (ACPAs), a hallmark of RA. Since then, it has been shown that antibodies to *P. gingivalis* are more common in serum from patients with RA compared to matched controls [5, 6]. In addition, the presence of anti-*P. gingivalis* antibodies has also been associated with ACPAs in individuals at increased risk of RA [7]. Furthermore, the disease-specific ACPAs [8], purified from RA serum, may cross-react with citrullinated *P. gingivalis* enolase [9] due to the unique property of *P. gingivalis* to express citrullinating enzymes, named *P. gingivalis*-derived peptidylarginine deiminase (PPAD), which may, together with host’s endogenous PADs, contribute to local citrullination [4, 10, 11]. Increased expression of human PAD2 and PAD4 enzymes has been observed in gingival tissue of patients with periodontitis and, both human PAD and bacterial enzyme PPAD activities have been reported to be elevated in both RA and non-RA patients with periodontitis [12, 13].

*Aggregatibacter actinomycetemcomitans* (*A. actinomycetemcomitans*), another Gram-negative periodontal bacteria, has long been associated with chronic and aggressive periodontitis [14], and has also been detected in gingival crevicular fluid of patients with RA [15]. Moreover, recently, the pore-forming leukotoxin (LtxA), secreted by *A. actinomycetemcomitans*, was shown to be capable of triggering the activation of endogenous PAD in neutrophils through the disruption of the cell membrane and increased cellular calcium influx [15], suggesting that this pathogen could be another candidate bacteria for gingival citrullination triggering autoimmunity in RA.

Citrullination of proteins is a posttranslational conversion of peptidylarginine to peptidylcitrulline by PAD enzymes that occurs naturally in different physiological processes, as well as in numerous pathological processes, including inflammation and autoimmunity. The periodontitis-associated pathogens *P. gingivalis* and *A. actinomycetemcomitans* have been reported to be involved in citrullination [11, 13, 15] but not their effects on citrullination and expression of endogenous PAD enzymes. Therefore, the aim of this study was to investigate the presence of citrullinated proteins and expression of endogenous PADs (PAD2 and PAD4) in relation to the periodontal pathogens *P. gingivalis* and *A. actinomycetemcomitans* leukotoxin in periodontal tissue of individuals with and without periodontitis as well as in that of patients with RA.

## Methods

### Collection of gingival biopsies

Gingival biopsies were collected from 15 patients with periodontitis (mean age 50 ± 19) and 15 individuals without periodontal disease, non-periodontitis, (mean age 43 ± 19). Inclusion criteria for the periodontitis groups were clinical signs of periodontitis at the site of biopsy collection, including radiographic bone resorption and pocket depth larger than 5 mm. Gingival tissue collected from control subjects were obtained from sites with no evidence of alveolar bone loss and pocket depth less than 3 mm [16, 17]. Subjects using anti-inflammatory drugs or having a systemic disease were excluded from the study. In addition, gingival tissues from four patients with both RA and periodontitis were also collected for this study. The Regional Ethics Review Board in Stockholm approved the study and each patient gave informed consent for the use of gingival tissue.

### Histology and immunohistochemistry staining

Gingival tissues were fixed in 4% neutral buffered formalin (Apoteket, Sweden) followed by standard dehydration and paraffin embedding procedures. Serial Sections (4 μm) were cut from the paraffin blocks using microtome. For histology, standard hematoxylin and eosin staining protocol was performed.

For immunohistochemistry, the sections were deparaffinised using xylene and rehydrated through a series of decreasing ethanol concentrations to Phosphate Buffered Saline (PBS). Next, endogenous blocking step was performed using 1% H_2_O_2_ (Merck, Germany) in PBS for 60 min. Antibodies against CD3, citrullinated epitopes, PAD2, PAD4, *P. gingivalis* and leukotoxins LktA or LtxA were incubated on the sections overnight. The Leukotoxins LtxA, from *A. actinomycetemcomitans*, and LktA, from *Mannheimia haemolytica*, were both used due to extensive amino acid homology [18]. Detection of horseradish peroxidase activity was performed using the VECTASTAIN ABC kit (Vector Laboratories, CA, USA) accordingly to the manufacturers’ protocol. The sections were counterstained with Mayer’s hematoxylin (HTX, Histolab Products AB, Sweden), dehydrated through a series of increasing ethanol concentration to xylene. Finally, sections were mounted with PERTEX^®^ (Histolab Products AB, Sweden). All steps were carried out at room temperature unless otherwise specified. As negative isotype controls following antibodies were used: for F95 purified mouse myeloma, IgM (Zymed, CA, USA); for PAD2, LktA and CD3 rabbit immunoglobulin fraction; for PAD4 mouse IgG1 and for *P. gingivalis* mouse IgG2b (all from Dako, Denmark).

For T cell marker CD3, sections were antigen retrieved in 10 mM Tris, 1 mM EDTA buffer solution pH 9.0 using microwave heating after deparaffinising and rehydrating. The staining procedure was performed as described above, but using PBS with 0.1% saponin for all steps. Sections were incubated with CD3 rabbit polyclonal antibody (1 µg/ml, A0452, Dako, Denmark). A blocking step with 1% normal goat serum (NGS, Dako, Denmark) was employed for 15 min prior to the secondary antibody incubation. Next, the biotinylated goat anti-rabbit IgG (1:1600, Vector Laboratories, CA, USA) was applied on the sections for 30 min.

Presence of citrullinated proteins was detected using mouse IgM monoclonal antibody clone F95, as previously described [19]. For detection of PAD2, rabbit polyclonal antibody and for PAD4, mouse monoclonal antibody isotype IgG2a was used for staining. Antigen retrieval step was performed for PAD4 staining in 10 mM citrate buffer pH 6.0 using the 2100 Antigen Retriever unit (Aptum Biologics Ltd, UK). The sections were blocked with 3% BSA-0.3% Triton X-100 in PBS for 30 min and followed by F95 (1:1500, Merck Millipore, MA, USA), PAD2 (0.2 µg/ml, ROI002, Cosmo Bio, Japan) and PAD4 (1 µg/ml, ab128086, Abcam, UK) incubation. An additional blocking step with 1% NGS for 15 min was performed prior to the 30 min of incubation with following biotinylated antibodies: goat anti-mouse IgM (1:250), goat anti-rabbit IgG (1:1600) and horse anti-mouse IgG (1:640) (all from Vector Laboratories, CA, USA).

The presence of *P. gingivalis* in the gingival tissue was detected using the in-house purified mouse monoclonal antibody, as described in the next section. The staining procedure was performed as to the mentioned CD3 staining protocol. Primary *P. gingivalis* antibody (1 µg/ml) was applied overnight. On the following day, the sections were blocked with 1% normal horse serum (Dako, Denmark) for 15 min before the secondary biotinylated horse anti-mouse IgG antibody (1:640, Vector Laboratories, CA, USA) was added for 30 min. The presence of leukotoxins was detected by rabbit polyclonal antibodies against *Mannheimia haemolytica* (*M. haemolytica*) LktA (0.4 µg/ml, ABIN2833367, Antibodies-Online, Germany) and in-house production rabbit polyclonal antibodies against *A. actinomycetemcomitans* leukotoxin LtxA (1:100, Umeå University, Sweden) [20]. The staining procedure was performed using Cell and Tissue Staining kit (CTS005, R&D Systems, MN, USA) and according to the manufacturer’s instruction.

### Production and purification of *P. gingivalis* monoclonal antibody

Hybridoma cell line HB-9968 producing mouse monoclonal antibody isotype IgG2b reactive to *P. gingivalis* was purchased from the American Type Culture Collection (ATCC), USA [21]. Cells were cultured in Dulbecco’s Modified Eagle’s Medium containing 4 mM l-glutamine. Briefly, a column containing Protein G Sepharose 4 Fast Flow (GE Healthcare, IL, USA) was pre-rinsed with 20 mM disodium hydrogen pH 7.0 and then conditioned cell culture medium was applied. Protein G bound antibody was eluted with 0.1 M glycine hydrochloride pH 2.7 and dialysed against PBS. The purified antibodies (1.12 mg/ml) were used for immunohistochemical analysis.

### Semi-quantitative evaluation of immunohistochemistry staining

Semi-quantitative evaluation of the stained gingival tissue sections was performed by three independent blinded observers using Nikon Eclipse E600 (Nikon Instruments Inc, Japan) and Olympus BX43F (Olympus Corporation, Japan) microscopes. The grade of inflammation was assessed in all the HTX stained gingival biopsies using a four point scale: 0 = no signs of inflammation, 1 = minimal inflammation, 2 = moderate inflammation and 3 = extensive inflammation. The degree of positively antibody-specific stained cells was evaluated using another four point scoring scale indicating: 0 = no positive cells, 1 = minimal presence, 2 = moderate amounts and 3 = high degree of stained cells [17, 22].

### Quantitative RT-qPCR

Total RNA was isolated from gingival tissue biopsies of subjects with periodontitis and helthy controls without periodontitis using the RNeasy Mini Kit (Qiagen, CA, USA) as previously described [17, 23]. The amount of total RNA was quantified using a Qubit spectrophotometer (Molecular Probes, OR, USA). cDNA synthesis was performed from 1 µg of total RNA per 20 µl of reaction using the iScript™ cDNA Synthesis Kit (BioRad, CA, USA), according to manufacturer’s instructions. The mRNA expression of *PADI2 and PADI4* was performed by quantitative reverse transcription PCR (RT-qPCR) using TaqMan Gene Expression Assays together with TaqMan Universal PCR Master Mix (Applied Biosystems, CA, USA). TaqMan probes were used as follows: *PADI2* (Hs00247108_m1), *PADI4* (Hs00202612_m1) and the housekeeping gene glyceraldehyde 3-phosphate dehydrogenase (*GAPDH*; Hs02758991_g1). All reactions were run in duplicates on the 7500 Fast Real-Time PCR system (Applied Biosystems, CA, USA). mRNA expression was calculated according to the ΔΔCt method, where the periodontitis samples were normalized to the healthy control samples and to corresponding *GAPDH* (reference gene) sample.

### Statistical analysis

Mann–Whitney U-test was used to analyse the differences between histological scores of inflammation, CD3-positive lymphocytes, expressions of citrullinated proteins, PAD2, PAD4, and the presence of *P. gingivalis* and leukotoxins in the two groups (periodontitis versus non-periodontitis) of participants. Percentages of positive samples were tested by means of Fisher’s exact test. Wilcoxon signed rank test was used to analyse RT-qPCR data. A *p*-value < 0.05 was considered significant.

## Results

### Presence of inflammatory cells and infiltrating lymphocytes in gingival tissue

**Fig. 1:**
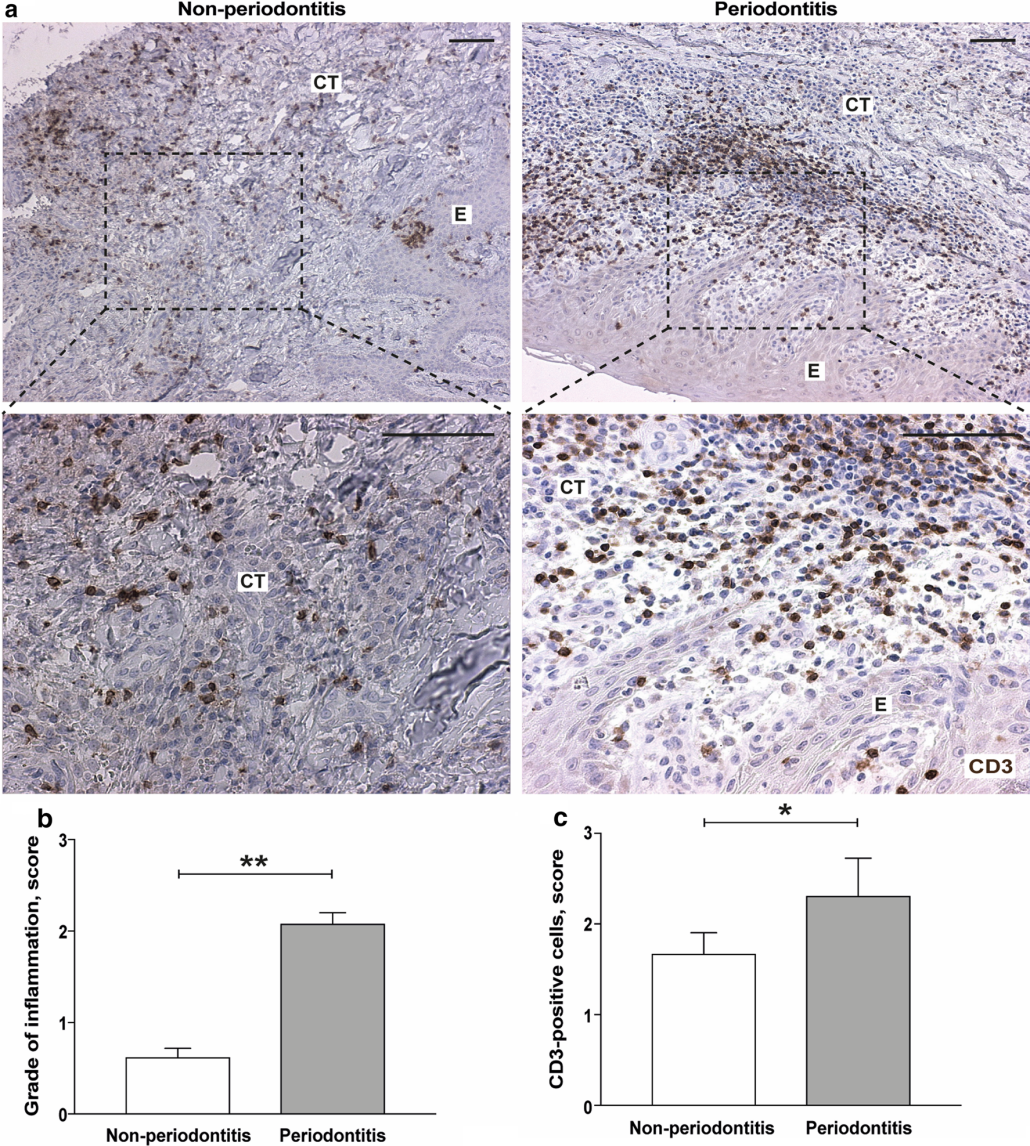
Evaluation of the gingival tissue inflammation in the patients with and without periodontitis. **a** Representative immunohistochemical staining of CD3-positive lymphocytes in gingival tissue samples obtained from patients with periodontitis and periodontally healthy controls (non-periodontitis). Magnification ×100 and ×2.5 zoom-in on the area of interest (scale bars 100 μm). **b** and **c** graphs show mean of **b** positive cell scores of inflammation (± SD) and **c** CD3-positive lymphocytes in tissue sections obtained from patients with periodontitis (n = 15) and non-periodontitis subjects (n = 15). *E* epithelium, *CT* connective tissue. *p < 0.05; **p < 0.01

The degree of inflammation was evaluated using CD3-stained gingival biopsies, obtained from patients with periodontitis and periodontally healthy controls (non-periodontitis) (Fig. 1a). High levels of inflammation were present in gingival tissue of individuals with periodontitis (100%, 15/15 subjects), whereas low levels were also detected in gingival tissue biopsies from patients without periodontitis (47%, 7/15). The semi-quantitative evaluation of HTX staining, revealed significantly (*p* < 0.01) higher degree of inflammation in periodontitis, with mean score 2.08 ± 0.22 as compared to non-periodontitis, with mean score 0.62 ± 0.18 (Fig. 1b). Similarly, the number of CD3-positive lymphocytes (mean score 2.31 ± 0.75 for periodontitis and 1.67 ± 0.42 for healthy controls) was significantly higher (*p* < 0.05) in gingival tissue of subjects with periodontitis, confirming the recruitment of T lymphocytes and higher degree of inflammation (Fig. 1c).

### Presence of citrullinated proteins in gingival tissue

**Fig. 2:**
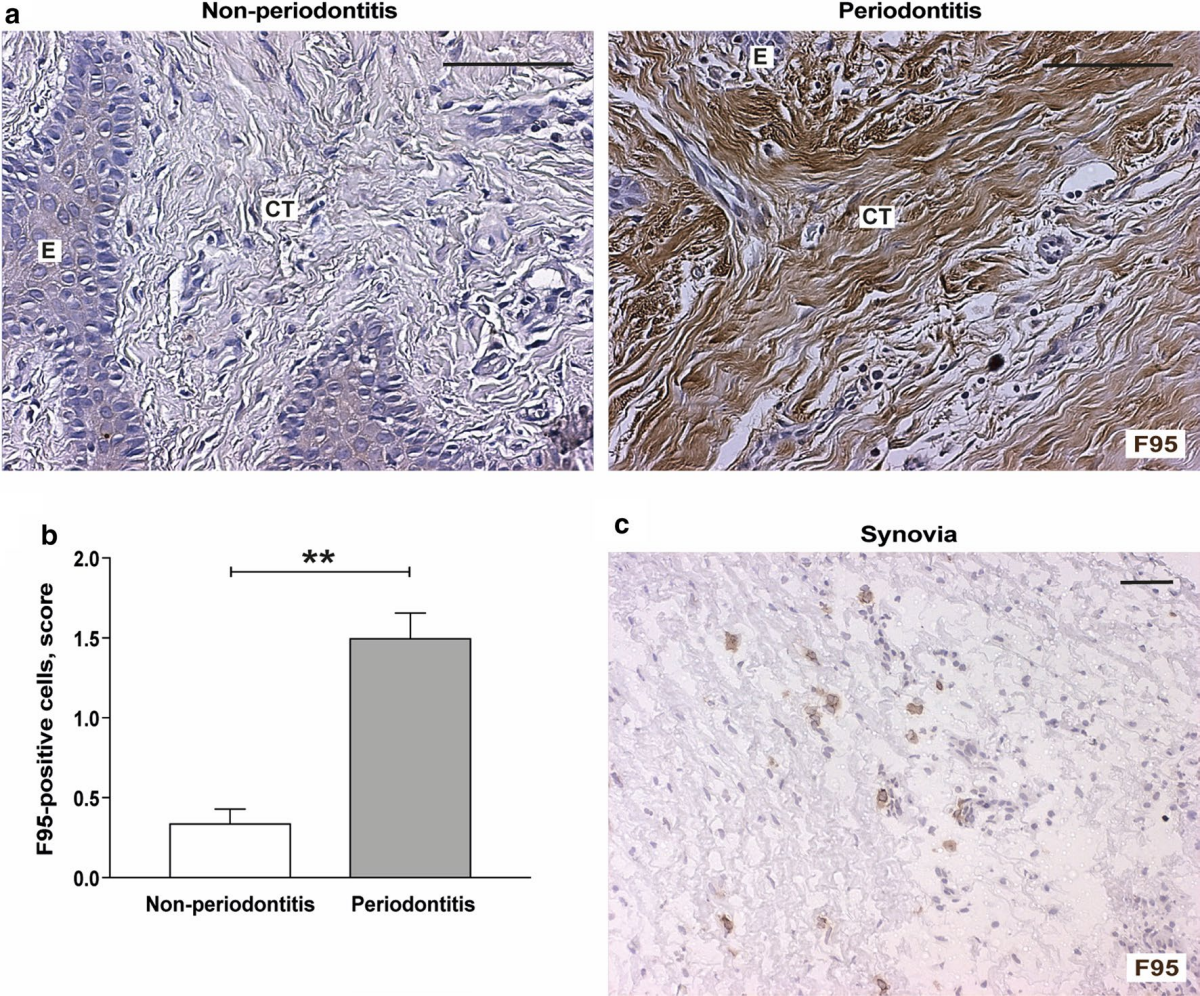
Expression of citrullinated proteins in gingival tissue samples of patients with and without periodontitis. **a** Representative immunohistochemical staining of citrullinated proteins in gingival tissue sections obtained from patients with periodontitis and periodontally healthy controls (non-periodontitis). Magnification ×250 (scale bars 100 μm). **b** Graph shows mean positive cell scores of citrullinated proteins (F95)-positive cells (± SD), stained in gingival tissue sections obtained from patients with periodontitis (n = 15) and non-periodontitis subjects (n = 15). *E* epithelium, *CT* connective tissue. **p < 0.01. **c** Immunohistochemical staining of synovial tissue used as positive control for citrullinated proteins. Magnification ×100 (scale bars 100 μm)

Presence of antibodies to citrullinated proteins is one of the hallmarks of autoimmunity in RA. During inflammation, citrullinated proteins can serve both as triggers of the production as well as targets of such antibodies. Therefore, we investigated the expression of citrullinated proteins in gingival tissues of healthy and periodontitis affected individuals using immunohistochemistry. These proteins were mainly detected within and in association with inflammatory cells but also in fibroblast-like cells and in the extracellular matrix of the connective tissue (Fig. 2a, Additional file 1: Figure S1). Citrullinated proteins were present in a majority of the gingival tissues of individuals with periodontitis (80%, 12/15 subjects), while only in a few of the healthy group (27%, 4/15). Furthermore, the semi-quantitative evaluation of cells, stained for citrullinated proteins, revealed significantly (*p* < 0.01) higher levels of citrullination in gingival connective tissue of periodontitis patients with mean score 1.50 ± 0.28 compared to control subjects with mean score 0.34 ± 0.16 (Fig. 2b). As to the presence of citrullination in the epithelial compartment, there were no significant differences between periodontitis (40%, 6/15) and healthy (33%, 5/15) groups. Synovial tissue from a patient with RA was used as a positive control (Fig. 2c), as increased protein citrullination has previously been associated with synovial inflammation [17, 22]. Staining with isotype-matched mouse antibody was negative in all tissues (figure not shown).

### PAD2 and PAD4 expressions in gingival tissue

**Fig. 3:**
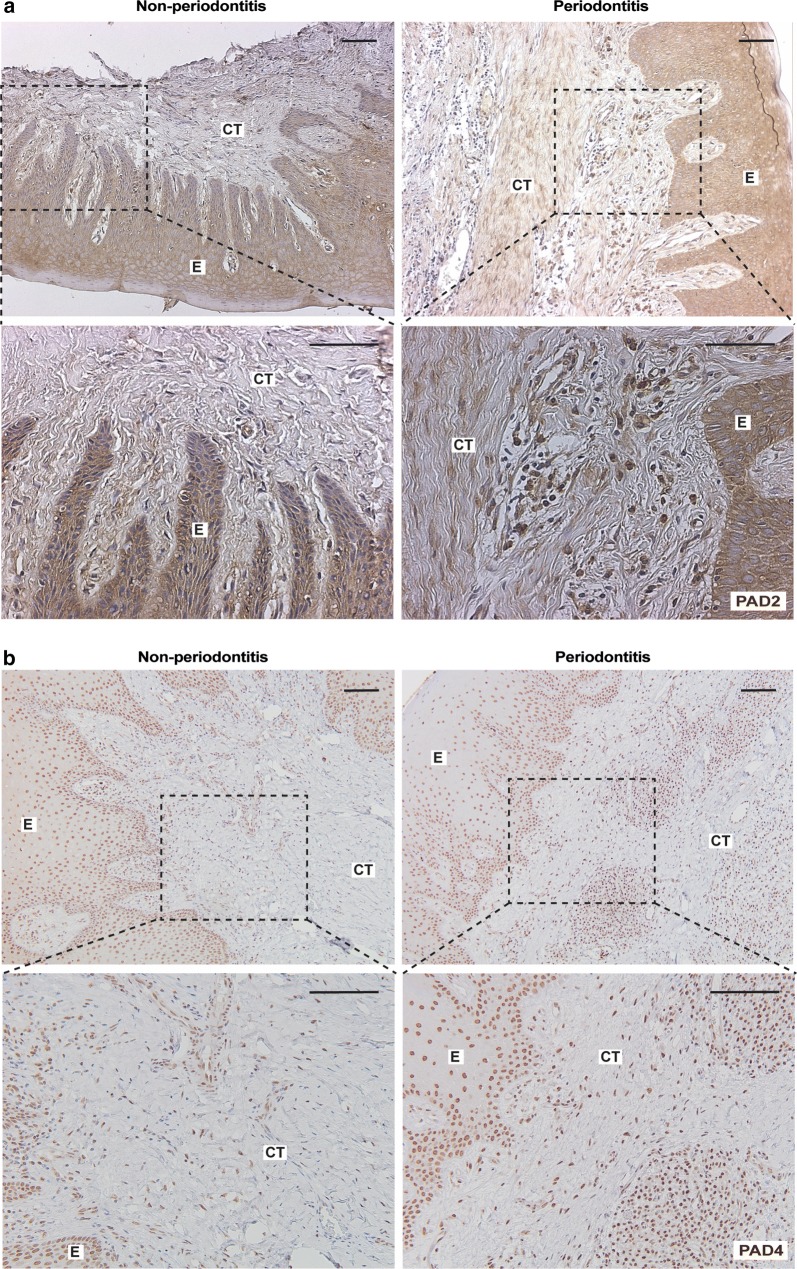
PAD2 and PAD4 expression in gingival tissue samples of patients with and without periodontitis. Gingival biopsies from patients with periodontitis (n = 15) and periodontally healthy controls without periodontitis (n = 15) were analysed using immunohistochemistry for **a** PAD2 and **b** PAD4 expression (representative images). Magnification ×100 and ×2 zoom-in on the area of interest (scale bars 100 μm). *E* epithelium, *CT* connective tissue

**Fig. 4:**
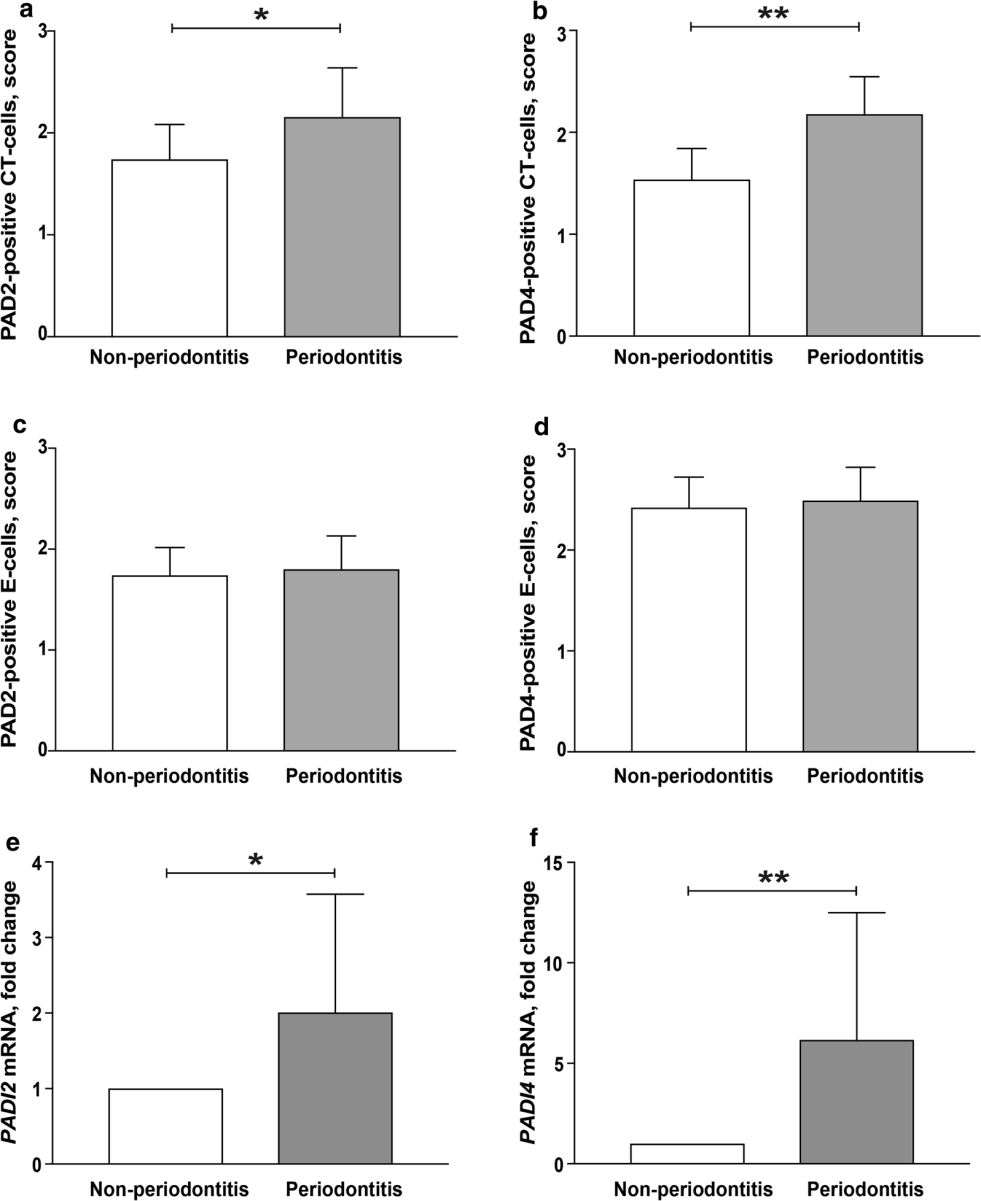
PAD2 and PAD4 protein and mRNA expression in gingival tissues of patients with and without periodontitis. Mean positive cell scores (± SD) for **a** and **c** PAD2 and **b** and **d** PAD4 in connective and epithelial tissue sections (respectively) from patients with periodontitis (n = 15) and subjects without periodontitis (n = 15). Gene expression of **e** *PADI2* and **f** *PADI4* in gingival tissue biopsies of patients with periodontitis (n = 13) and without periodontitis (n = 11) using RT-qPCR. The mRNA expression is shown as fold change of periodontitis samples relative to healthy controls and calculated according to the ΔΔCt method, where periodontitis samples were normalized to the non-periodontitis samples and to corresponding *GAPDH* sample. *p < 0.05; **p < 0.01

Protein citrullination occurs both naturally and in inflammatory conditions due to endogenous peptidylarginine deiminases (PADs). Our results, obtained from immunostaining of gingival biopsies with antibodies against PAD2 (Fig. 3a) and PAD4 (Fig. 3b), revealed the expression of these proteins in gingival tissues of patients with periodontitis, as well as healthy controls without periodontitis. However, the protein expressions of both PAD2 (mean score 2.16 ± 0.87 for periodontitis and 1.74 ± 0.62 for healthy controls) and PAD4 (mean score 2.18 ± 0.66 for periodontitis and 1.54 ± 0.55 for healthy) were significantly higher (*p* < 0.05 and *p* < 0.01, respectively) in the gingival connective tissue of patients with periodontitis compared to healthy controls (Fig. 4a, b, respectively). Furtermore, PAD2 and PAD4 staining could be observed in macrophage-like cells of the inflammatory infiltrate, in fibroblast-like and in the endothelial cells. No significant differences were observed in the expressions of PAD2 (mean score 1.80 ± 0.60 for periodontitis and 1.74 ± 0.50 for healthy controls) or PAD4 (mean score 2.49 ± 0.60 for periodontitis and 2.42 ± 0.55 for healthy controls) in the epithelium compartment between these two groups (Fig. 4c, d). Similarly to the protein expression of PAD2 and PAD4, the mRNA expression of *PADI2* and *PADI4,* assessed by qPCR analysis, was also significantly higher (p < 0.05 and p < 0.01, respectively) in the gingival tissue of patients with periodontitis compared to healthy controls without periodontitis (Fig. 4e, f).

### Presence of *P. gingivalis* and leukotoxins in gingival tissue of patiens with periodontitis

**Fig. 5:**
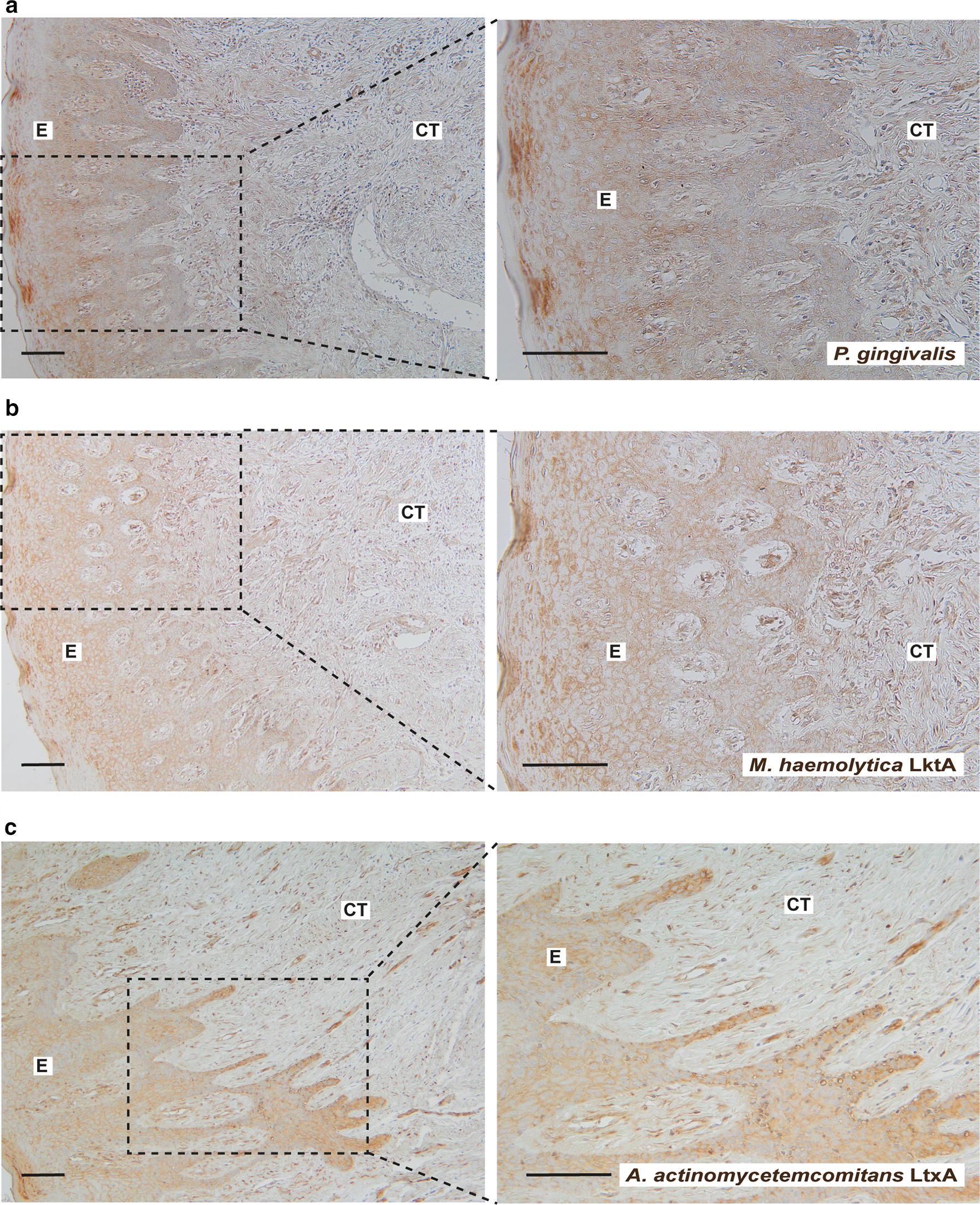
Detection of *P. gingivalis* and leukotoxins of *M. haemolytica* and *A. actinomycetemcomitans* in gingival biopsies from patients with periodontitis. Representative immunohistochemistry images of gingival biopsies (n = 15), stained for **a** *P. gingivalis*, **b** *M. haemolytica* LktA and **c** *A. actinomycetemcomitans* LtxA are shown. Magnification ×100 and ×2 zoom-in on the area of interest (scale bars 100 μm). *E* epithelium, *CT* connective tissue

The oral pathogens *P. gingivalis* and *A. actinomycetemcomitans* are known to be associated with periodontitis, and have also been linked to RA [15, 24, 25]. In the current study both periodontitis (73%, 11/15 subjects) and healthy (60%, 9/15) gingival biopsies were positively stained with antibodies against *P. gingivalis* (Fig. 5a, Additional file 2: Figure S2, respectively), with no significant difference between periodontitis and non-periodontitis gingival tissue samples. Mean score for periodontitis was 1.31 ± 0.28 in the connective tissue and 1.02 ± 0.24 in the epithelium and for healthy 1.17 ± 0.28 in the connective tissue and 1.55 ± 0.21 in the epithelium. Furthermore, there was no correlation between the presence of *P. gingivalis* and the expression of citrullinated proteins or the citrullinating enzymes PAD2 and PAD4.

Recently, periodontitis has been proposed to be linked to RA by the actions of leukotoxin A (LtxA), produced by the oral pathogen *A. actinomycetemcomitans*. In this study we therefore also investigated the expression of LtxA in gingival tissue biopsies. To detect the presence of leukotoxin in the epithelium and connective tissue regions of gingival tissues, we used antibodies against *A. actinomycetemcomitans* LtxA and *M. haemolytica* LktA, as these two lymphotoxins share extensive amino acid homology [18]. Strong staining with the *M. haemolytica* LktA antibody was observed in the epithelium region of both periodontitis (Fig. 5b) and healthy gingival tissues (figure not shown) with no significant differences between the groups (mean score 2.83 ± 0.32 and 2.58 ± 0.70, respectively) (*p* = 0.275). Moreover, there were no differences in the gingival connective tissue of periodontitis patients (mean score 2.43 ± 0.6) compared to controls without periodontitis (mean score 2.07 ± 0.9). Similar results were observed, when using a second polyclonal antibody, produced against *A. actinomycetemcomitans* LtxA, with regard to staining pattern and intensity (Fig. 5c). Further analysis of the gingival sections showed that citrullinated proteins, *P. gingivalis* as well as leukotoxins were detected in the gingival epithelium and connective tissue, where leukocytes, fibroblast-like and endothelial cells stained positive (Fig. 5).

### Presence of *P. gingivalis* and leukotoxins in gingival tissue of patients with periodontitis and RA

**Fig. 6:**
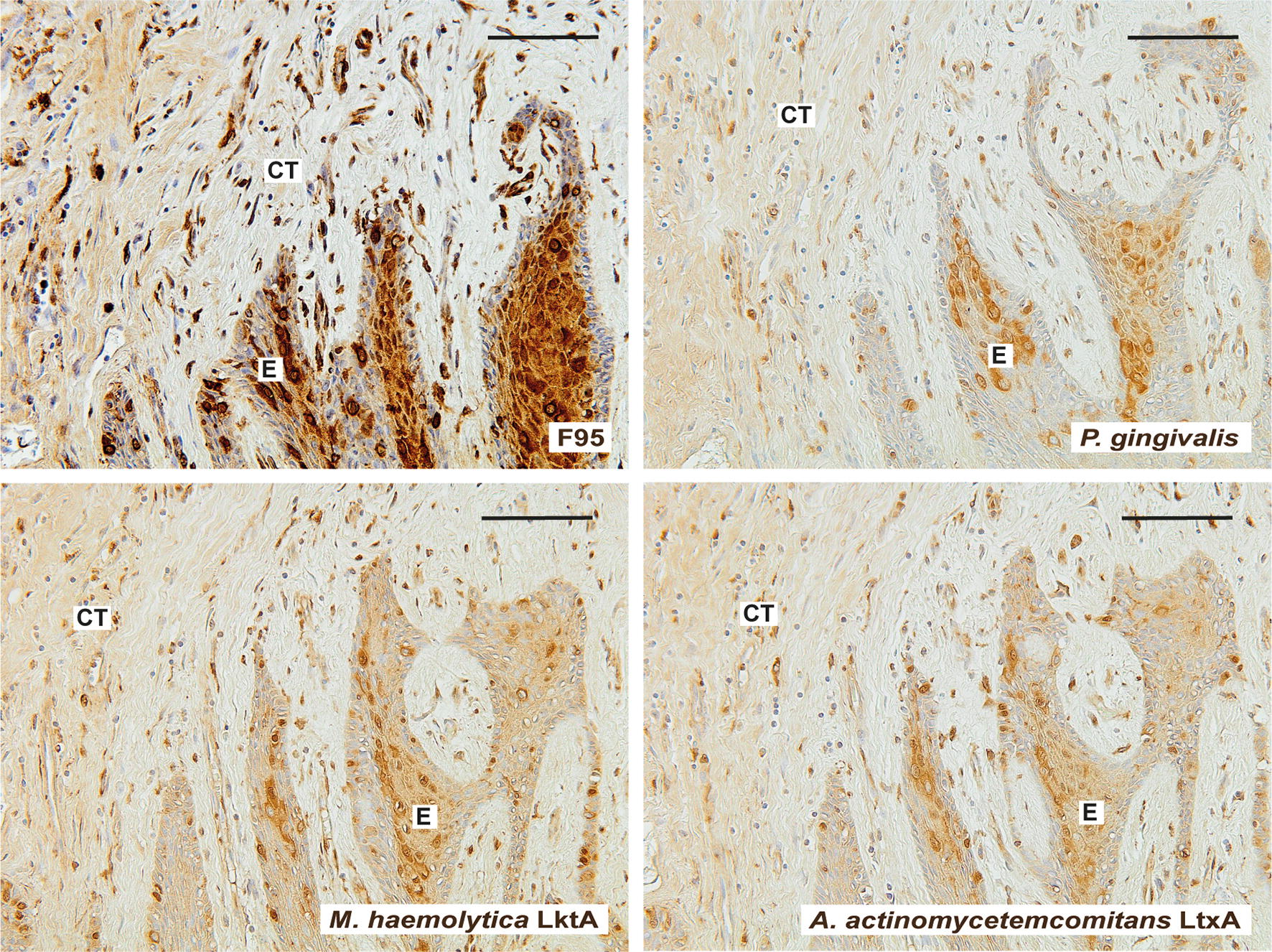
Detection of citrullinated proteins, *P. gingivalis* and leukotoxins of *M. haemolytica* and *A. actinomycetemcomitans* in gingival biopsies from patients with periodontitis and RA. Representative immunohistochemistry images of gingival biopsies (n = 4), stained for citrullinated proteins, *P. gingivalis*, *M. haemolytica* LktA and *A. actinomycetemcomitans* LtxA in gingival tissue samples are shown. Magnification ×200 (scale bars 100 μm). *E* epithelium, *CT* connective tissue

We also tested the presence of citrullinated proteins, *P. gingivalis* and leukotoxins of *M. haemolytica* and *A. actinomycetemcomitans* in gingival tissue of four patients, diagnosed with both periodontitis and RA. These limited number of gingival biopsies, similar to the results obtained from gingival tissue of patients with periodontitis, were also stained positively for citrullinated proteins, *P. gingivalis*, and both leukotoxins (Fig. 6). Additional analysis of the biopsies revealed that citrullinated proteins, *P. gingivalis* and leukotoxins were localised to the epithelium of gingival and connective tissue, and that leukocytes, fibroblast-like and endothelial cells stained positive (Fig. 6).

## Discussion

Several studies have shown clinical and epidemiological associations between periodontitis and RA, although the strength, temporal relationship and biological explanations to these associations remain unclear [26, 27]. The main finding in the present study was that both citrullination and expression of endogenous PADs (PAD2 and PAD4), both at mRNA and protein level, are increased in gingival connective tissue of patients with periodontitis compared to periodontally healthy controls independently of the presence of the periodontal pathogen *P*. *gingivalis* or leukotoxin of *A. actinomycetemcomitans*.

Anti-citrullinated protein antibodies are present in about two-thirds of all patients with RA, but rare in the non-RA population [28]. It has been suggested that immunity towards citrullinated proteins may be triggered in genetically predisposed individuals by an increased expression of citrullinated proteins in the inflamed sites of the body, for example in the lungs of smokers or the periodontium of patients with periodontitis [12, 29–31]. This immunity may subsequently contribute to the development of chronic inflammatory processes in the joint, where citrullination is also present [22, 31–33]. In case of periodontitis, it has been established that periodontal pathogens initiate a local host response in the periodontal pocket, involving recruitment of inflammatory cells, with increased apoptosis and necrosis of neutrophils in the gingival connective tissue, which may contribute to deamination of arginine residues and hypercitrullination of proteins [34]. In agreement with this, our results showed increased levels of citrullination in gingival connective tissue of patients with periodontitis compared to periodontally healthy controls. On the contrary, in the gingival epithelium, where citrullination occurs as a physiological process [12], the levels of citrullination did not differ between the periodontitis and non-periodontitis groups.

It has been shown that bacterial PAD enzymes, expressed by the periodontitis-associated bacteria *P*. *gingivalis*, is capable of citrullination of endogenous bacterial, as well as human proteins, suggesting a role for this pathogen in this aetiological model [25, 35]. Apart from *P. gingivalis*, another periodontal bacteria, *A. actinomycetemcomitans*, has also been suggested as a potential trigger of gingival citrullination through the actions of a pore-forming leukotoxins [15]. Hence, in order to elucidate the involvement of these oral pathogens in generating citrullinated proteins in gingival tissue we have investigated citrullination and expression of human PADs, in relation to the presence of *P. gingivalis* and leukotoxins of *A. actinomycetemcomitans* and *M. haemolytica*, in gingival tissue of patients with periodontitis and periodontally healthy controls. In the current study, the presence of *P*. *gingivalis* was comparable in gingival tissue samples from patients with periodontitis and periodontally healthy controls, while increased protein citrullination and human PAD expression were observed in periodontitis-affected tissue, which is in line with previous studies [12, 30, 36, 37]. However, we could not observe a correlation between the presence of *P. gingivalis* and citrullinated proteins, or the expression of endogenous PADs (i.e. PAD2 and PAD4), suggesting that the increased citrullination, observed in this limited number of patients, is caused by the actions of host PAD enzymes, rather than *P. gingivalis*-produced PAD. Notably, increased expression of human PAD enzymes has been reported previously at the site of inflammation including gingival tissue and is likely to contribute to the increased levels of citrullination [12, 30, 36, 37]. Our study was not designed to determine whether citrullination in the gingival tissue of patients with periodontitis was caused by human PAD enzymes or enzymatic activity of PPADs was involved. Moreover, Laugisch et al. [13] have shown that PPAD activity is higher in gingival crevicular fluid samples of both RA and non-RA patients with periodontitis compared to those without periodontitis.

The periodontal bacteria *A. actinomycetemcomitans* has been shown to induce hypercitrullination in neutrophils by activating human PAD enzymes through the actions of leukotoxins [15]. In the current study the presence of leukotoxins was detected in gingival tissue from patients with periodontitis. However, there was no association between the presence of leukotoxins and the expression of citrullinated proteins or human PAD enzymes in gingival biopsies from patients with periodontitis. Thus, our results may suggest that the increased citrullination in the gingival connective tissue in periodontitis is independent of the presence of the periodontal pathogen *P. gingivalis* and leukotoxin of *A. actinomycetemcomitans*. One possible explanation for this might be that the progression of the polymicrobial disease periodontitis is dependent not only on the quantity of the periodontal pathogens but also on quality, local host response as well as other risk factors such as smoking and genetic predisposition [38]. Notably, the gram-negative anaerobic periodontal pathogens *P. gingivalis* and *A. actinomycetemcomitans* are commensal bacteria commonly present in gingival tissue, as also observed in our study. The pathogenicity of periodontitis is initiated via colonization of “keystone pathogens”, such as *P. gingivalis*—which even in low numbers—can increase the virulence of the entire community by communicating with other commensal organisms, promoting dysbiosis, the transition to pathogenicity and, thereby, indirectly ensuing the inflammation [34, 39, 40]. Another explanation might be that different strains of *P. gingivalis* are known to differ in their virulence factors [41], including PAD activity [42] and the aetiological model linking *P. gingivalis* to ACPA-positive RA depends on the bacteria’s ability to citrullinate, which we have not investigated in our study. In addition, other bacteria may also be involved, as the oral microbiome *Cryptobacterium curtum* was recently suggested to be involved in the production of autoantigenic citrullinated peptides in RA [43]. Furthermore, the *P. gingivalis*- and *A. actinomycetemcomitans*-independent increase of citrullination and PAD expression may also be a consequence of cellular hypoxia, which has been associated with inflammatory disorders such as periodontitis and RA [44, 45]. This anaerobic microenvironment promotes citrullination and expression of PAD through activation of hypoxia-inducible factor-1α (HIF1α) [46], a proinflammatory key transcription factor [45], reported to be induced by hypoxia in human synoviocytes and in gingival fibroblasts [44, 46].

Our study mainly investigated non-RA study individuals, which may differ from RA patients with regard to oral pathogens, PAD enzymes, and the oral “citrullinome”. Unfortunately, the number of RA patients in our study (n = 4) was too low to make any relevant comparisons, therefore future studies with larger sample size should address potential differences between systemically healthy patients with periodontitis and RA patients with periodontits. Importantly, increased anti-*P. gingivalis* antibody levels have been reported in RA (in particular ACPA-positive RA), compared to systemically healthy patients with periodontitis [47], and increased anti-LtxA antibody levels have been described in RA, in relation to ACPAs and HLA-DR4 alleles [15]. Thus, the possibility that the co-occurrence of RA and periodontitis might have a common denominator associated with citrullination and anti-citrulline immunity deserves a series of additional studies, directed toward understanding the specificity and the pathogenicity of citrullination and anti-citrulline immunity, in periodontitis and RA. Nevertheless, this is to our knowledge the first study investigating the presence of citrullinated proteins and expression of PAD2 and PAD4 in relation to the oral pathogens *P. gingivalis* and leukotoxin of *A. actinomycetemcomitans*, in gingival tissue biopsies, however the results should be confirmed in additional studies.

## Conclusions

Taken together, our results confirm previous findings, that chronic gingival inflammation is associated with increased local protein citrullination as well as PAD2 and PAD4 expression in gingival tissue of patients with periodontitis, and additioanally, that this process appears to be independent of the presence of *P. gingivalis* and *A. actinomycetemcomitans* leukotoxin.

## Additional files

**Additional file 1: Figure S1.** Expression of citrullinated proteins. Another representative example of citrullinated proteins staining in gingival tissue sections obtained from patients with periodontitis and periodontally healthy controls (non-periodontitis). Magnification 250x (scale bars 100 μm).

**Additional file 2: Figure S2.** Detection of *P. gingivalis* and leukotoxins of *M. haemolytica* and *A. actinomycetemcomitans* in gingival biopsies from healthy patients without periodontitis. Representative immunohistochemistry images of gingival biopsies are shown. Magnification 100x and 2x zoom-in on the area of interest (scale bars 100 μm). E = Epithelium; CT = Connective Tissue.

## Abbreviations

*A. actimomycetemcomitans*: *Aggregatibacter actinomycetemcomitans*
ACPA: anti-citrullinated protein/peptide antibodies
LktA: *M. haemolytica* leukotoxin A
LtxA: leukotoxin A
*M. haemolytica*: *Mannheimia haemolytica*
PAD2 and PAD4: peptidylarginine deiminases 2 and 4
*P. gingivalis*: *Porphyromonas gingivalis*
PPAD: *P. gingivalis*-derived peptidylarginine deiminase
RA: rheumatoid arthritis
