# Supplementary figures and images for: Increased citrullination and expression of peptidylarginine deiminases independently of P. gingivalis and A. actinomycetemcomitans in gingival tissue of patients with periodontitis
Source: J Transl Med. 2018 Jul 31;16:214. doi: 10.1186/s12967-018-1588-2 (PMC6069803; doi:10.1186/s12967-018-1588-2)

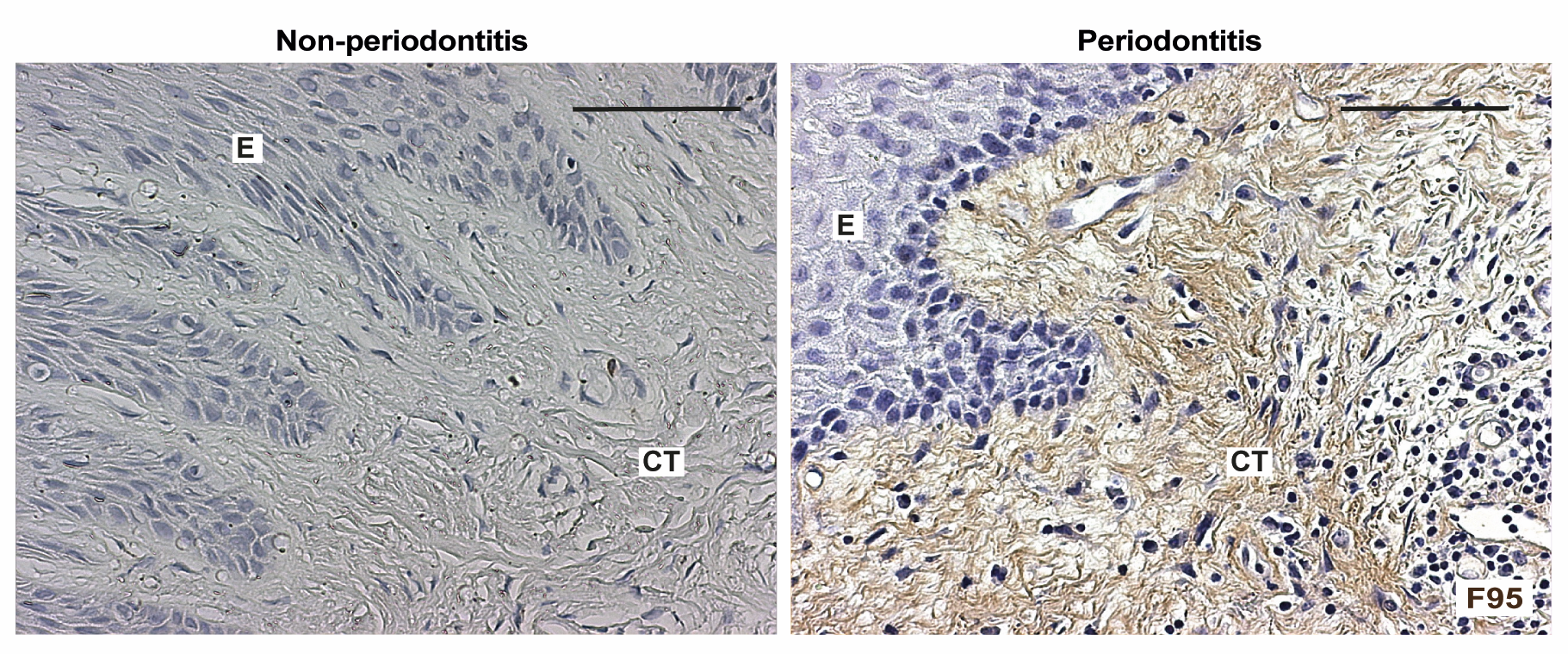

Supplement: Supplementary file 1 — Additional file 1: Figure S1. Expression of citrullinated proteins. Another representative example of citrullinated proteins staining in gingival tissue sections obtained from patients with periodontitis and periodontally healthy controls (non-periodontitis). Magnification 250x (scale bars 100 μm). [file 12967_2018_1588_MOESM1_ESM.tif]

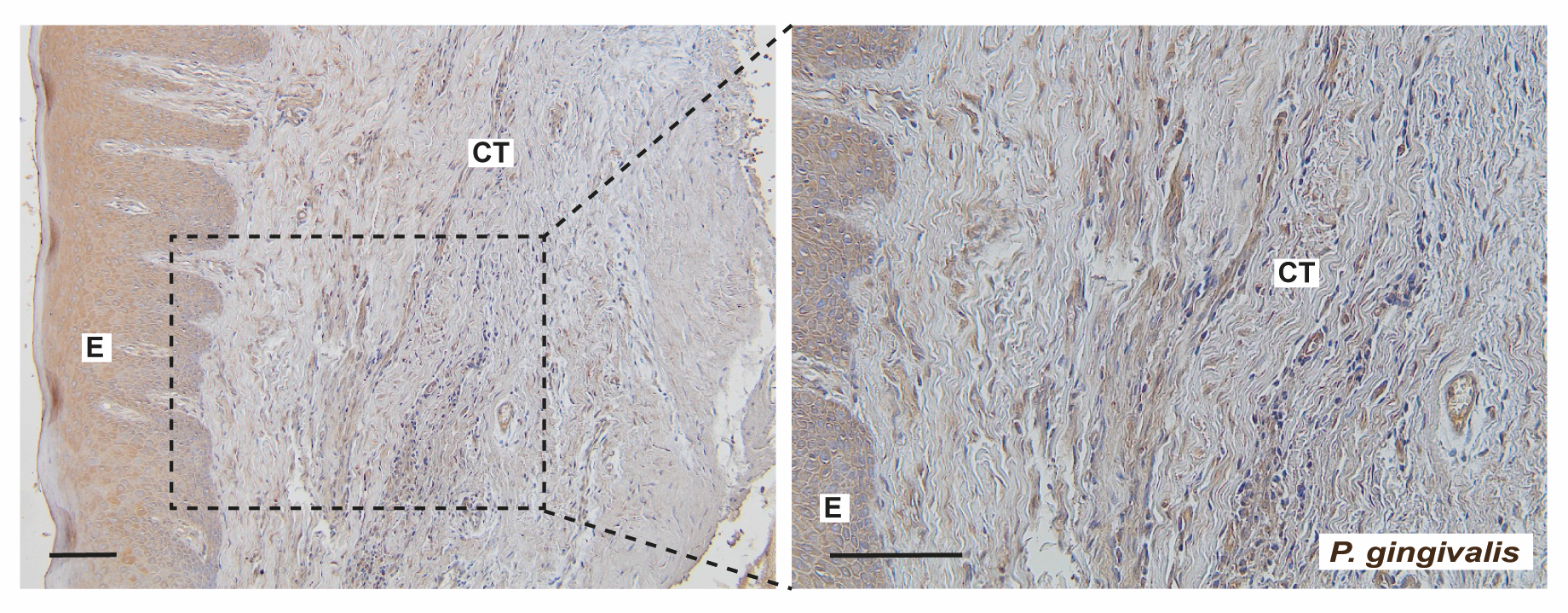

Supplement: Supplementary file 2 — Additional file 2: Figure S2. Detection of P. gingivalis and leukotoxins of M. haemolytica and A. actinomycetemcomitans in gingival biopsies from healthy patients without periodontitis. Representative immunohistochemistry images of gingival biopsies are shown. Magnification 100x and 2x zoom-in on the area of interest (scale bars 100 μm). E = Epithelium; CT = Connective Tissue. [file 12967_2018_1588_MOESM2_ESM.tif]
